# Heterogeneous Learning of Functional Clustering Regression and Application to Chinese Air Pollution Data

**DOI:** 10.3390/ijerph20054155

**Published:** 2023-02-25

**Authors:** Tingting Wang, Linjie Qin, Chao Dai, Zhen Wang, Chenqi Gong

**Affiliations:** 1 School of Statistics, Huaqiao University, Xiamen 361021, China; 2 Department of Economics, Xiamen University, Xiamen 361005, China; 3 College of Mathematics and Statistics, Chongqing University, Chongqing 401331, China

**Keywords:** functional data analysis, heterogeneity learning, clustering regression, meteorological data

## Abstract

Clustering algorithms are widely used to mine the heterogeneity between meteorological observations. However, traditional applications suffer from information loss due to data processing and pay little attention to the interaction between meteorological indicators. In this paper, we combine the ideas of functional data analysis and clustering regression, and propose a functional clustering regression heterogeneity learning model (FCR-HL), which respects the data generation process of meteorological data while incorporating the interaction between meteorological indicators into the analysis of meteorological data heterogeneity. In addition, we provide an algorithm for FCR-HL to automatically select the number of clusters, which has good statistical properties. In the later empirical study based on PM_2.5_ concentrations and PM_10_ concentrations in China, we found that the interaction between PM_10_ and PM_2.5_ varies significantly between regions, showing several types of significant patterns, which provide meteorologists with new perspectives to further study the effects between meteorological indicators.

## 1. Introduction

Environmental pollution is a hot global issue and has been given high attention by all governments. Among air pollutants, particulate matter (PM) poses the greatest risk to human health [1]. The Organization for Economic Cooperation and Development (OECD) estimates that air pollution will be the leading environmental cause of death by 2050. Therefore, it is important to mine the regional heterogeneity of air pollution and its internal patterns. Meteorological data such as temperature, humidity, atmospheric pressure, and pollutant concentrations are continuously changing in the atmosphere at a specific location. Unfortunately, we are unable to obtain the original curves of continuous data directly. The usual approach is to sample at a given time interval, thus obtaining time-series type data in discrete cases. Obviously, no matter how intensive our sampling time is, there is no way to avoid information loss. When performing heterogeneity mining, it is necessary to calculate the distance between meteorological data from different regions. There are generally two types of method for discrete type of meteorological data. The first one is to extract representative observation statistics from continuous time by series, e.g., mean, variance, etc. [2,3], and the second is to treat time series data according to the ordinary high-dimensional Euclidean space. The former method further produces information loss from data processing, while the latter maintains the full picture of discrete information, but there is a problem of “curse of dimensionality” due to the high dimensionality when calculating the distance of sample observations. However, the use of functional data analysis (FDA) for meteorological data can avoid these problems and bring more advantages in data processing.

First of all, FDA respects the data generation process more. It converts discrete data into functional data by interpolation or smoothing [4]. The advantage of this treatment is that it retains as much information as possible about the variation of the sample in the time domain, while also preserving the characteristics of the curve fluctuations. In addition, with Functional Principal Component Analysis (FPCA), functional data objects can be projected from time domain data to frequency domain data, providing a frequency domain perspective for time series analysis. Intuitively, the meteorological time series is decomposed into a combination of functional components, which is also consistent with the characteristics of meteorological data. For example, temperature is influenced by diurnal and seasonal variations, pollutant concentration is influenced by seasonal variations, traffic peaks, and other cyclical factors. In particular, meteorological data is often a combination of multiple trends over time. After FPCA, we can calculate the distance between sample observations based on the finite component scores, which can effectively avoid the problem of dimensional curse. Therefore, mining the heterogeneity and internal pattern of meteorological data under the perspective of FDA has natural advantages.

Heterogeneous learning is achieved through clustering methods. Clustering is the typical method to mining heterogeneity. Clustering algorithms are a classical class of unsupervised learning algorithms that cluster samples based on the similarity of their observations. There exists a wide range of applications in the mining of meteorological data, such as analysis of spatial and temporal variation of pollutants, air quality monitoring and optimization of monitoring network, and correlating pollutant concentrations with specific synoptic conditions [4]. The commonly used clustering methods to study meteorological data are Partitioning clustering [5,6,7], Hierarchical clustering [8,9,10], Fuzzy clustering [11,12,13] and Model-based clustering such as the EM method, SOM method, etc. [14,15].

The above applications and models all have a common defect, that is, they are all clustered directly based on the data itself, ignoring the potential structural information between the related data, and the heterogeneity between the clusters is insufficiently described. In fact, there are complex correlations between various air pollutants. For example, nitrogen oxides are correlated with each other [16], and there are complex correlations between the concentrations of PM_10_ and PM_2.5_ [17]. The correlation between meteorological data has the potential to optimize the clustering algorithm. Therefore, from the idea of clustering regression, we want to excavate the potential relationship between different meteorological indicators and use it as auxiliary information to guide the clustering.

Cluster regression was first mentioned by Späth [18], which has given rise to new ideas and vitality in the era of big data. Joki et al. [19] introduced the support vector machine model in machine learning into CLR (Cluster-wise linear regression), transformed the problem into an unconstrained non-smooth optimization problem, and designed a method based on an incremental algorithm and double beam method combined with the DC optimization method. Numerical experiments verify the reliability and effectiveness of the method. The results show that the method after adding a support vector machine optimizes the partitioning effect when outliers in the data. Amb et al. [20] designed a CLR algorithm based on DCA (difference of convex algorithm) and incremental method and used the quasi-Newton method to solve the problem. They found that the new method can effectively solve the CLR problem under large-scale data from the evaluation of synthetic data and real data. Da Silva and de Carvalho [21] proposed the W-CLR (Weighted Cluster-wise Linear Regression) model, which solves the possible overfitting problem of the original model and can better describe the linear relationship of subspace samples. Experiments on the W-CLR synthetic dataset and benchmark datasets validate the effectiveness of the method. In terms of the application of the model, Bagirov et al. [22] selected the monthly rainfall data from 1889 to 2014 in eight different geographic locations in Australia and proposed a clustered linear regression (CLR) method for monthly precipitation forecasting. The results show that the method has advantages over models such as multiple linear regression, neural networks, and support vector machines. Torti et al. [23] studied the heterogeneity problem from the perspective of CLR based on EU mask trade data, achieved the selection of the optimal number of clusters and the best combination through a two-step method, and obtained the optimal stable solution. However, the above research is still at the level of linear regression. With increasingly complex data and interrelationships, simple linear regression can hardly describe the potential connections between the data accurately.

This paper combines the dual advantages of FDA perspective with cluster regression and proposes a functional clustering regression heterogeneity learning method (FCR-HL). In summary, FCR-HL has the following three advantages: (1) clustering from FDA perspective, which is more suitable for the generation process of meteorological data, and greatly reduces the information loss problem of the original data. (2) The auxiliary information (i.e., the regression relationship between air pollutants) is incorporated into the clustering process to optimize the clustering results. In addition, the regression patterns within each group can be automatically identified. (3) We develop an adaptive selection process of the number of clusters, effectively avoiding the limitation of manual setting. The clustering results have a direct impact on the subsequent studies. The model we constructed can optimize the clustering results while mining the heterogeneity patterns of different groups, and thus has practical significance and application value.

## 2. Methods

The FCR-HL model mainly solves four problems: (1) the optimization problem of clustering: partitioning data into different clusters with the perspective of regression can incorporate more information to attain a better clustering effect, and how to solve the optimization problem is the key point. (2) Parameter estimation problem: the parameters in the regression that explain the impact of the covariate on the response variable need to be estimated within each cluster. (3) Clustering number estimation that decides how many clusters are needed is an important part for the model. (4) Iterative algorithm: it is difficult to solve the problem of both partition and parameter estimation simultaneously; our model gives an iterative process to solve the three problems mentioned above. The following will explain the solutions to the four problems, respectively.

### 2.1. Clustering Optimization and Parameter Estimation

Ramsay and Dalzell [24] proposed functional data analysis, which uses non-parametric ideas to fit data, and can effectively capture the continuous characteristics of data. In the functional data analysis, the functional regression model is an effective and convenient method. This paper mainly focuses on one typical functional regression models, that is, the covariates are functional data, and the response variables are scalar types, which have a functional covariate and scaler response variable:

(1) $$Y_{i}=\alpha_{0}+\int X_{i}\left(t\right)\alpha_{1}\left(t\right)dt+e_{i},i=1,2,\ldots ,n,t\in \left[0,T\right]$$

where the response variable $Y_{i}$ is a scalar and the vector expression is $Y={\left(Y_{1},Y_{2},\ldots ,Y_{n}\right)}^{\prime }$, *n* is the observation number, and the covariate variable $X_{i}\left(t\right),t\in \left[0,\;T\right]$ represents ith functional trajectory that has a bounded upper limit T. Assuming $e_{i}\sim \mathrm{iid}\;N\left(0,\sigma^{2}\right)$, the Karhunen-loeve expansion can be used for the functional covariates to obtain Equation (2):

(2) $$X_{i}\left(t\right)=u\left(t\right)+\overset{\infty }{\underset{k=1}{\sum }}\xi_{ik}\varphi_{k}\left(t\right)$$

where u(t)=E[X(t)] represents the mean function of the covariate, and$\;\varphi_{k}\left(t\right)$ is the eigenfunction corresponding to the $k^{th}$ largest eigenvalue $\lambda_{k}$ of the covariance G(s,t)=Cov(X(t),X(s)), the eigenfunctions are orthogonal to each other, and satisfy $\int \varphi_{k}^{2}\left(t\right)dt=1$ and $\int \varphi_{k}\left(t\right)\varphi_{l}\left(t\right)dt=0,\;k\neq l$. Using the functional principal component analysis (FPCA), $\xi_{ik}$ named as the functional principal component scores of $X_{i}\left(t\right)-u\left(t\right)$ in the direction of $\varphi_{k}\left(t\right)$ are obtained, which satisfy $E\left[\xi_{ik}\right]=0$ and $Var\left[\xi_{ik}\right]=\lambda_{k}$. According to Formula (2), Formula (1) can be rewritten as:

(3) $$Y_{i}=\alpha_{0}+\int \left[u\left(t\right)+\overset{\infty }{\underset{k=1}{\sum }}\xi_{ik}\varphi_{k}\left(t\right)\right]\alpha_{1}\left(t\right)dt+e_{i}\approx \beta_{0}+\overset{K}{\underset{k=1}{\sum }}\beta_{k}\xi_{ik}+e_{i}$$

where $\beta_{0}=\alpha_{0}+\int u\left(t\right)\alpha_{1}\left(t\right)dt$, $\beta_{k}=\int \varphi_{k}\left(t\right)\alpha_{1}\left(t\right)dt$, the mean function u(t) of $X_{i}\left(t\right)$ is mapped to the constant parameter $\beta_{0}$, and $\varphi_{k}\left(t\right)$ are mapped to the parameter $\beta_{k}$. In other words, the parameter $\beta_{0}$ includes the mean value of $Y_{i}$ when $X_{i}\left(t\right)=0$ and the information of the mean trend of $X_{i}\left(t\right)$, and the parameter $\beta_{k}$ stands for the effect of the kth deviations of $X_{i}\left(t\right)$ on $Y_{i}$. In this way, the auxiliary information between the covariate and the response variable is reflected in the parameter $\beta_{k}$. This paper builds the FCR-HL model based on the auxiliary information to cluster the data.

For Equation (3), the summation term is truncated at K, which is determined using the AIC criterion Li, et al. [25], which is to estimate the optimal K, which is given by minimizing the sum of the pseudo-Gaussian loglikelihood and K. Note $Y={\left(Y_{1},Y_{2},\ldots ,Y_{n}\right)}^{T}$, $\xi_{i}={\left(1,\xi_{1},\xi_{2},\ldots ,\xi_{K}\right)}^{T},\;\xi ={\left(\xi_{1},\xi_{2},\ldots ,\xi_{n}\right)}^{T},\;\beta ={\left(\beta_{0},\beta_{1},\beta_{2},\ldots ,\beta_{K}\right)}^{T},\;e={\left(e_{1},e_{2},\ldots ,e_{n}\right)}^{T}$, we rewrite Formula (2) in a matrix expression:

(4) Y=ξβ+e

The advantage of using FPCA technology is that the infinite-dimensional functional data can be converted into low-dimensional data, and then it helps to construct a linear regression model relating to the principal component scores. On one hand, this method can reduce the computational difficulty and the algorithm complexity due to the dimensional curse. On the other hand, it can preserve the nonlinear characteristics in the covariate, which are utilized for the regression analysis. At the same time, the principal component scores estimated by FPCA have good statistical characteristics, especially the unbiasedness and consistency, which are helpful for inferring the subsequent parameter estimations discussed later.

The goal of clustering optimization in this paper is to cluster data from the perspective of the regression hyperplane. The FCR-HL model mainly has two steps of iterations: First, obtaining the parameter estimates under the given partition. Second, clustering samples based on the parameter estimates. According to the two-step iterative algorithm, the optimal regression clustering results can be found.

Firstly, given a partition, the parameters are estimated from the perspective of the regression hyperplane and with the data that has been partitioned. Compared with the random partition, it takes the relationship between the covariate and response variable as an auxiliary information for clustering. The parameters can be estimated with greater accuracy once the partition has deduced the heterogeneity between the data. It is assumed that the samples from the same partition have the following relationship:

(5) $$y_{i_{m}}=\xi_{i_{m}}^{\prime }\beta_{m}+e_{m},e_{m}\sim N\left(0,\sigma_{m}^{2}\right),i_{m}\in C_{m},m=1,2,\ldots ,M$$

where $C=\left\{C_{1},\;C_{2},\ldots ,\;C_{M}\right\}$ represent the sub-populations and $\overset{M}{\underset{m=1}{\sum }}\left|C_{m}\right|=n$ where $\left|C_{m}\right|$ is the samples size of the cluster $C_{m}$, and M is the number of clusters, which may grow with sample size, $y_{i_{m}}$ are the observed response data belonging to the cluster $C_{m}$, $\xi_{i_{m}}$ are the vector scores derived from the observed functional covariate $x_{i}\left(t\right)$ belonging to the cluster $C_{m}$, and $\beta_{m}={\left(1,\beta_{m1},\;\beta_{m2},\ldots ,\beta_{mK}\right)}^{T}$ are the coefficients of the cluster $C_{m}$.

In (5), it is necessary to first solve the unknown functional principal component scores, and then we can estimate the parameter $\beta_{m}$. It should be noted that the estimate of the functional principal component scores can directly affect the result of the parameter estimates, considering that the PACE (principal component analysis through conditional expectation) method proposed by Yao, et al. [26] is unbiased and consistent estimation method for the functional principal component scores. The PACE method gives the estimators ${\hat{\xi }}_{ik}=\hat{E}(\xi_{ik}|X_{i})$ where $X_{i}={\left(X\left(t_{i1}\right),\;X\left(t_{i2}\right),\ldots ,X\left(t_{in_{i}}\right)\right)}^{\prime }$ and $\xi_{ik}$ and $X\left(t_{ij}\right)$ are jointly Gaussian. Then, the PACE method is used to estimate the functional principal component scores and the mean function according to Formula (2), by which the principal component score estimates $\hat{\xi }$ and the estimation of the mean function $\hat{u}\left(t\right)$ have the following convergence properties:

(6) $$sup_{t\in Τ}\left|\hat{u}\left(t\right)-u\left(t\right)\right|=O_{p}\left(\frac{1}{\sqrt{n}h_{u}}\right)$$

(7) $$\underset{n\to \infty }{\lim}\hat{\xi }=E[\xi |X]\;in\;probability$$

where $\hat{u}\left(t\right)$ is obtained by the local liner smoother, and $h_{u}$ is the bandwidth used in the local linear smoother. Formulas (6) and (7) show that $\hat{u}\left(t\right)$ converges to u(t) and $\hat{\xi }$ are unbiased estimates for ξ when n→∞, which are the good statistical characteristics mentioned before. Thus, ξ can be replaced by the estimates $\hat{\xi }$ as a new regression model shown in Formula (8):

(8) $$y_{i_{m}}={\hat{\xi }}_{i_{m}}^{\prime }\beta_{m}+e_{i_{m}},e_{i_{m}}\sim N\left(0,\sigma_{i_{m}}^{2}\right),i\in C_{m},m=1,2,\ldots ,M$$

Based on Formula (8), the log-likelihood function can be shown in Formula (9):

(9) $$logL_{n}\left(K,C,\left(\beta_{1},\sigma_{1}^{2}\right),\ldots \left(\beta_{M},\sigma_{M}^{2}\right)\right)=-\frac{1}{2}\overset{M}{\underset{m=1}{\sum }}\underset{i\in C_{m}}{\sum }\left(log2\pi +log\sigma_{i_{m}}^{2}+\frac{{\left(y_{i_{m}}-{\hat{\xi }}_{i_{m}}^{\prime }\beta_{m}\right)}^{2}}{\sigma_{i_{m}}^{2}}\right)$$

It is difficult to obtain the optimal partition and the estimates of the unknown parameters in (9) just by maximizing $logL_{n}\left(M,C,\left(\beta_{1},\sigma_{1}^{2}\right),\ldots \left(\beta_{M},\sigma_{M}^{2}\right)\right)$. Thus, an iterative method is proposed. Firstly, the optimization objective of clustering, fixing $\beta_{m}$ at ${\hat{\beta }}_{m}$ and $\sigma_{i_{m}}^{2}$ at ${\hat{\sigma }}_{m}^{2}$, is to maximize the log-likelihood function when the observation data $\left(y_{i},\;x_{i}\left(t\right)\right)$ belongs to the cluster:

(10) $$\hat{C_{m}}=argmax_{1\leq m\leq M}\left(logL_{n}\left(M,C_{m},\left({\hat{\beta }}_{m},{\hat{\sigma }}_{m}^{2}\right)\right)\right)\;=argmax_{1\leq m\leq M}\left\{-\frac{1}{2}\left(log2\pi +log{\hat{\sigma }}_{m}^{2}+\frac{{\left(y_{i}-{\hat{\xi }}_{i_{m}}^{\prime }{\hat{\beta }}_{m}\right)}^{2}}{{\hat{\sigma }}_{m}^{2}}\right)\right\}\;\propto argmin_{1\leq m\leq M}\left\{log{\hat{\sigma }}_{m}^{2}+\frac{{\left(y_{i_{m}}-{\hat{\xi }}_{i_{m}}^{\prime }{\hat{\beta }}_{m}\right)}^{2}}{{\hat{\sigma }}_{m}^{2}}\right\}\;$$

To solve Formula (10), the parameter estimations $\left({\hat{\beta }}_{m},{\hat{\sigma }}_{m}^{2}\right)$ needs to be obtained. The idea is to maximize the log-likelihood function of the data within the class. Formula (11) is the log-likelihood function of the data $i\in C_{m}$:

(11) $$logL_{n}\left(M,C_{m},\left(\beta_{m},\sigma_{m}^{2}\right)\right)=\underset{i\in C_{m}}{\sum }(-\frac{1}{2}\left(log2\pi +log\sigma_{m}^{2}+\frac{{\left(y_{i}-{\hat{\xi }}_{i_{m}}^{\prime }\beta_{m}\right)}^{2}}{\sigma_{m}^{2}}\right)$$

Then, the parameters ${\hat{\beta }}_{m}$ are obtained according to the maximum likelihood estimation:

(12) $${\hat{\beta }}_{m}=argmax_{\beta_{m}}\left\{\underset{i\in C_{m}}{\sum }(-\frac{1}{2}\left(log2\pi +log\sigma_{i_{m}}^{2}+\frac{{\left(y_{i_{m}}-{\hat{\xi }}_{i_{m}}^{\prime }\beta_{m}\right)}^{2}}{\sigma_{i_{m}}^{2}}\right)\right\}={\left({\hat{\xi }}_{i_{m}}^{\prime }{\hat{\xi }}_{i_{m}}\right)}^{-1}\left({\hat{\xi }}_{i_{m}}^{\prime }y_{i_{m}}\right)$$

(13) $${\hat{\sigma }}_{m}^{2}=\frac{\sum_{i\in C_{m}}{\left(y_{i}-{\hat{\xi }}_{i_{m}}^{\prime }{\hat{\beta }}_{m}\right)}^{2}}{\hat{n_{m}}}$$

(14) $$\hat{n_{m}}=\left|\hat{C_{m}}\right|$$

where $\hat{n_{m}}$ represents the sample size of the cluster $\hat{C_{m}}$ and ${\hat{\sigma }}_{m}^{2}={\hat{\sigma }}_{i_{m}}^{2}$ for simplification. Then, parameter estimations ${\hat{(\beta }}_{m},{\hat{\sigma }}_{m}^{2},\hat{C_{m}})$ are brought into Formula (9) to obtain the log-likelihood function of the complete data:

(15) $$logL_{n}\left(M,C,\left({\hat{\beta }}_{1},{\hat{\sigma }}_{1}^{2}\right),\ldots \left({\hat{\beta }}_{M},{\hat{\sigma }}_{M}^{2}\right)\right)=-\frac{1}{2}\overset{M}{\underset{m=1}{\sum }}\underset{i\in C_{m}}{\sum }\begin{array}{c} \left(log2\pi +log{\hat{\sigma }}_{m}^{2}+\frac{{\left(y_{i_{m}}-{\hat{\xi }}_{i_{m}}^{\prime }{\hat{\beta }}_{m}\right)}^{2}}{{\hat{\sigma }}_{m}^{2}}\right) \end{array}$$

When fixing the partition $C_{m}$, the ${\hat{\beta }}_{m}$ and ${\hat{\sigma }}_{m}^{2}$ are the maximum likelihood estimators of the regression within the cluster, as shown in (12) and (13). When fixing ${\hat{\beta }}_{m}$ and ${\hat{\sigma }}_{m}^{2}$, the, the likelihood function will be maximized if the test data belongs to the cluster $C_{m}$. It is noted that the log-likelihood function is a monotonically increasing function, so it can reach the local maximum if a limited number of iterations are carried out alternatively. Furthermore, the parameter estimates derived from this optimization also have good statistical characteristics. First, the principal component scores obtained by FPCA are obtained by mapping the information of the data itself to the direction of the principal component. $\hat{\xi }$ are the unbiased estimates of ξ. Thus, $\hat{\xi }$ and ξ can be considered as non-random variables for the response variable Y, and the maximum likelihood estimation can give estimates having good statistical characteristics, for example, the unbiasedness:

(16) $$E\left(\hat{\beta_{m}}|X\right)=E\left(E\left(\hat{\beta_{m}}|\hat{\xi }\right)|X\right)=\beta_{m}$$

(17) $$Var\left(\hat{\beta_{m}}|\hat{\xi }\right)=E\left({\hat{\beta_{m}}}^{2}|\hat{\xi }\right)-{\left(E\left({\hat{\beta }}_{m}|\hat{\xi }\right)\right)}^{2}=\sigma^{2}{\left({\hat{\xi }}^{T}\hat{\xi }\right)}^{-1}$$

where variance of ${\hat{\beta }}_{m}$ can be used to verify the significance of the parameter. Because only the variance of ${\hat{\beta }}_{m}$ is estimated correctly, the significance results of the parameter estimates are reliable.

From Formulas (6), (7), (16), and (17), it can be known that the $\hat{\beta_{m}}$ converges to $\beta_{m}$ in probability. Therefore, it can be ensured that the obtained optimal number of clusters converges to the real number with probability 1 when data is clustered from the perspective of regression hyperplane.

In addition, it is also noted that the estimations using maximum likelihood are based on the classical assumption that the error term in Formula (8) obeys independently and identically normal distribution. Once the assumption is broken, the maximum likelihood estimation results are problematic. Thus, when it comes to the data which violates the independently and identically normal error distribution, a robust estimation (M-estimation) scheme, a generalized maximum likelihood estimation method is given. A special case of M-estimation is the Huber distribution, which has a normal distribution at the origin and an exponential distribution at the tail. The parameter estimation can be obtained according to the Huber distribution:

(18) $${\hat{\beta }}_{m}=argmin\left\{\underset{i\in C_{m}}{\sum }\rho_{c}\left(y_{i_{m}}-{\hat{\xi }}_{i_{m}}^{\prime }\beta_{m}\right)\right\}$$

(19) $$\rho_{c}\left(t\right)=\{\begin{array}{c} \frac{1}{2}t^{2},\left|t\right|<c\\ c\left|t\right|-\frac{1}{2}c^{2},\left|t\right|\geq c \end{array}$$

where $\rho_{c}\left(t\right)$ is the error function of the Huber distribution, and c is a fixed constant. Given parameter estimates ${\hat{\beta }}_{m}$, the optimal objective function for clustering can be obtained from sample observations $\left(y_{i_{m}},x_{i_{m}}\left(t\right)\right)$:

(20) $$\hat{C_{m}}=argmin\left\{\rho_{c}\left(y_{i_{m}}-{\hat{\xi }}_{i_{m}}^{\prime }\beta_{m}\right)\right\}$$

This function $\rho_{c}\left(t\right)$ is also strictly monotonically increasing.

The partition above is under the condition of a given number of clusters, so the next step is to give an estimation method for the number of clusters.

### 2.2. Estimation of the Optimal Number of Clusters

After estimating model parameters and optimizing the clustering scheme, we need to discuss the estimation of the optimal number of clusters. In this paper, the information criteria is used as the clustering loss function among the iterative algorithm. Then, we can simultaneously update the identification of heterogeneity in clusters and the optimal number of clusters.

After using the FPCA, the sample data is $\left\{\left(y_{1},{\hat{\xi }}_{1}\right),\left(y_{2},{\hat{\xi }}_{2}\right),\ldots ,\left(y_{n},{\hat{\xi }}_{n}\right)\right\}$. According to the previous analysis, it is assumed that the sample is composed of M sub-populations, and the characteristics of each population are represented by the regression hyperplane determined by the parameters.

Denote the partition $\left\{C=C_{m},m=1,2,\ldots ,M\right\},\;C_{m}≜\left\{m_{1},m_{2},\ldots ,m_{n_{m}}\right\}$, and obtain the regression model of each subpopulation is:

(21) $$Y_{C_{m}}={\hat{\xi }}_{C_{m}}^{\prime }\beta_{m}+e_{C_{m}}$$

(22) $$e_{C_{m}}\sim N\left(0,\sigma^{2}I_{n_{m}}\right)$$

(23) $$n_{m}=\left|C_{m}\right|$$

where $n_{m}$ is the sample size of cluster $C_{m}$ and $n=\overset{M}{\underset{m=1}{\sum }}n_{m}$, $Y_{C_{m}}={\left(y_{m_{1}},y_{m_{2}},\ldots ,y_{m_{n_{m}}}\right)}^{\prime },\;{\hat{\xi }}_{C_{m}}={\left({\hat{\xi }}_{m_{1}},{\hat{\xi }}_{m_{2}},\ldots ,{\hat{\xi }}_{m_{n_{m}}}\right)}^{\prime }$ is the response variable and principal component scores belonging to $C_{m}$, respectively, and ${\hat{\xi }}_{m_{j}}={\left({\hat{\xi }}_{m_{j1}},{\hat{\xi }}_{m_{j2}},\ldots ,{\hat{\xi }}_{m_{jK}}\right)}^{\prime }$ for $j=1,2,\ldots ,n_{m}$, and $I_{n_{m}}$ is a $n_{m}\times n_{m}$ identity matrix. Notice that ${\hat{\xi }}_{C_{m}}$ is a $K\times n_{m}$ matrix, both $Y_{C_{m}}$ and $e_{C_{m}}$ are $n_{m}\times 1$ vectors. The estimation of the number of clusters adopts the information criterion method based on the maximum likelihood estimation proposed by Shao and Wu [27], which is denoted as LS-C and can be obtained by:

(24) $$D_{n}\left({\hat{C}}_{M}\right)=\underset{C_{M}}{\min}\overset{M}{\underset{m=1}{\sum }}Y_{C_{m}}-{\hat{\xi }}_{C_{m}}^{\prime }{\hat{\beta }}_{m}{}^{2}+q\left(M\right)A_{n}$$

where ${\hat{\beta }}_{m}$ is estimated by the maximization likelihood estimation in this case, q(M) is a strictly increasing function of M and q(M)=MK generally, and $A_{n}\propto \log\left(n\right)$ or $A_{n}\propto loglog\left(n\right)$. The first part is the residual sum of squares and the second part is a penalty function relating to M and n. At the same time, Shao and Wu [27] have proved that the estimate derived by $D_{n}\left({\hat{C}}_{M}\right)$ will converge to the correct number of regression hyperplanes (or the number of the clusters) with probability 1 when the sample size is large enough (n→∞). It is noted that the LS-C is based on the maximum likelihood estimation. Again, a robust estimation for the case that does not have an independently and identically normal error distribution. Rao, et al. [28] constructed the robust information criterion denoted as RM-C:

(25) $$R_{n}\left({\hat{C}}_{M}\right)=\underset{C_{M}}{\min}\overset{M}{\underset{m=1}{\sum }}\underset{i\in C_{m}}{\sum }\rho_{c}\left(Y_{C_{m}}-{\hat{\xi }}_{C_{m}}^{\prime }{\hat{\beta }}_{m}\right)+q\left(M\right)A_{n}$$

where ${\hat{\beta }}_{m}$ is obtained by using the M-estimation in this case, and both q(M) and $A_{n}$ are same with Formula (24). By minimizing the information criteria LS-C or RM-C when the error distribution is independently identical normal or not, the number of clusters and the partition can be obtained. The advantage of the information criteria LS-C and RM-C is that the estimated number of clusters converges to the real number when the sample size is large enough, and the details can be referenced in Shao and Wu [27] and Rao, Wu and Shao [28].

### 2.3. Iterative Algorithm Design

In the FCR-HL model proposed in this paper, parameter estimation, clustering optimization, and the estimation of the number of clusters are all continuously updated in the iterative process, and this section will explain the iterative algorithm.

First, the residual sum of squares obtained based on maximum likelihood estimation within the cluster is recorded as RSS (residual squares sums), and the residual sum of squares obtained based on M-estimation is recorded as RRSS (robust residual squares sums):

(26) $$RSS\left(C_{M},\beta_{1},\beta_{2},\ldots ,\beta_{M}\right)=\overset{M}{\underset{m=1}{\sum }}Y_{C_{m}}-{\hat{\xi }}_{C_{m}}^{\prime }{\hat{\beta }}_{m}{}^{2}$$

(27) $$RRSS\left(C_{M},\beta_{1},\beta_{2},\ldots ,\beta_{M}\right)=\overset{M}{\underset{m=1}{\sum }}\rho_{c}\left(Y_{C_{m}}-{\hat{\xi }}_{C_{m}}^{\prime }{\hat{\beta }}_{m}\right)$$

Then, within the cluster RSS at each candidate M is calculated for the least square regression or the RRSS for the M-estimation based regression to approximate the local minimization, and determine the optimal cluster number by the information criteria LS-C or the RM-C, respectively.

In addition, the regression-based cluster method is easily affected by the initial partition. The global minimum of the information criterion or its good approximation can be achieved when using a good initial partition. Thus, it is necessary to determine the initial partition $C^{0}$. Based on the idea proposed in Qian, et al. [29], we extend it to handle the functional data. The following table Algorithm 1 shows the iterative initial partition algorithm.
**Algorithm 1** An iterative algorithm for initial partition.Step 1: Using the FPCA on X(t) to estimate the functional principal component score $\hat{\xi }$. Step 2: Through mapping the mean function u(t) and the basis function φ(t) to the parameter β, respectively, we build a functional regression model where the functional principal component scores are the covariates. Step 3: Parameters are estimated by the maximum likelihood estimation or robust estimation and based on the whole data. Step 4: (1) Set a distance threshold d and a sample size constant c. (2) For l=1, we calculate the distance between the point and the regression hyperplane obtained in Step 3. If the distance is less than the threshold d, then the point is partitioned into $C_{0,1}$, otherwise the point is partitioned into $C_{0,1}^{c}$, where $\left|C_{0,1}\right|>c,\;\;\left|\;C_{0,1}^{c}\right|>c$, otherwise go to Step 5. (3) For l=l+1, a point in the dataset $\cap_{i=1}^{l}C_{0,i}{}^{c}$, we estimate the parameters again and calculate the new distance. If the distance is less than d, the point is partitioned into $C_{0,l+1}$, otherwise into $C_{0,l+1}^{c}$, where $\left|C_{0,l+1}\right|>c,\left|C_{0,l+1}^{c}\right|>c$, otherwise go to Step 5. Step 5: Obtain the initial partition $C_{0}=\left\{C_{0,1},\ldots ,C_{0,l},\cap_{i=1}^{l}C_{0,i}{}^{c}\right\}$. 

It should be noted that the constants c and d are set based on the data. The initial partition is an iterative hierarchical binary clustering method, which adopts a regression model such as the least square regression in each iteration. The regression is robust, having a high breakdown threshold; thus, the Algorithm 1 is highly likely to produce a reasonable initial partition. After the initial partition, the iterative algorithm of the FCR-HL model are shown in the table Algorithm 2:
**Algorithm 2** The Partition iteration algorithm based on the initial partition.Step 1: Let s=1, we calculate the $RSS_{0}$ or $RRSS_{0}$ of the initial partition $C_{0}$, and the parameter estimation $\hat{\beta }$. Step 2: Let s=s+1, we calculate the RSS or RRSS of the data $\left(y_{i},{\hat{\xi }}_{i}\right)$ in $C_{0,\;i}$ and in $C_{0,j^{\prime }},j\neq j^{\prime }$, respectively, and we can obtain the $RSS_{min}$ or $RRSS_{min}$, where $RSS_{min}$ < $RSS_{0}$ or $RRSS_{min}$ < $RRSS_{0}$. Then the updated partition is $C_{j}=C_{j}+\left\{(y_{i},x_{i}\left(t\right)\right\},\;C_{i}=C_{i}-\left\{(y_{i},x_{i}\left(t\right)\right\}$, and let $RSS_{0}=RSS_{min}$ or $RRSS_{0}=RRSS_{min}$.Step 3: Continue to iterate Step 2 until $RSS_{0}$ or $RRSS_{0}$ no longer drops. 

In summary, the parameter estimations $\left\{{\hat{\beta }}_{m},{\hat{\sigma }}_{m}^{2},\hat{\;M}\right\}$ and the partition ${\hat{C}}_{M}=\left\{{\hat{C}}_{1},{\hat{\;C}}_{2},\ldots ,{\hat{\;C}}_{M}\right\}$ are updated in the iterative algorithm. Finally, the final regression clustering result can be obtained.

In the simulation analysis and the empirical data analysis, the K-means method has been used as a comparison model as it is a representative cluster method which only utilizes the distance between observations themselves. Our model emphasizes the importance of the auxiliary information between the response and covariate and cluster data from the regression perspective to dig the heterogeneity.

## 3. Results

### 3.1. Data Simulation

#### 3.1.1. Model Comparison Based on Heterogeneity Partitioning

We simulate data from three different groups that satisfies $Y_{ij}=\alpha_{0}^{j}+\int X_{ij}\left(t\right)\alpha_{1}^{j}\left(t\right)dt+\varepsilon_{i},\;i=1,2,\ldots ,500;\;j=1,2,3$. The number of units is 500 for each group and t is uniformly designed on [0,1]. Firstly, the functional covariate is generated by $X_{ij}\left(t\right)=\overset{6}{\underset{k=1}{\sum }}\xi_{ik}\varphi_{k}\left(t_{ij}\right)$ where $\xi_{ik}\sim N\left(0,1\right)$ and they are independent with each other k, and $\varphi_{k}\left(t\right)$ are the cubic spline basis functions. Then, the different coefficients in three groups are simulated by $\alpha_{1}^{1}\left(t\right)=\varphi_{1}\left(t\right)-\varphi_{2}\left(t\right),\;\alpha_{1}^{2}\left(t\right)=10\varphi_{1}\left(t\right)+7\varphi_{2}\left(t\right),\;\alpha_{1}^{3}\left(t\right)=-4\varphi_{1}\left(t\right)$, where $\varphi_{1}\left(t\right)=\sqrt{2}\sin\left(2\pi t\right),\;\varphi_{2}\left(t\right)=\sqrt{2}\cos\left(4\pi t\right)$.

**Figure 1:**
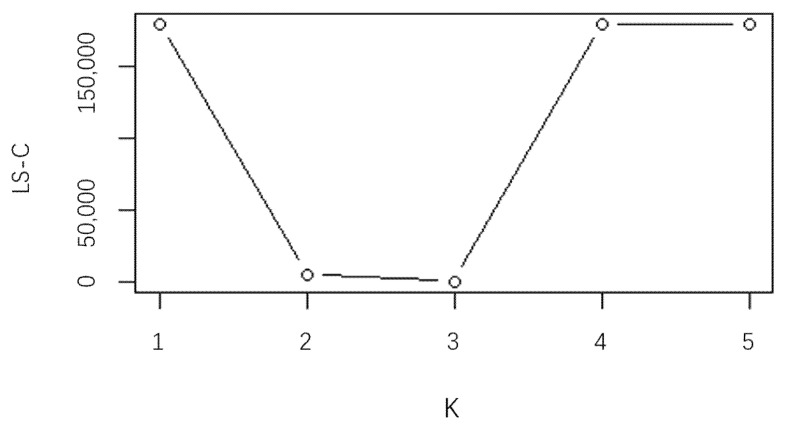
LS-C values over the candidate *K* for the simulation data.

After obtaining the functional principal component scores “Score1” and “Score2” of $X_{j}\left(t\right)$ that are treated as the new explanatory variables in the regression clustering model, our model can reduce the regression analysis from the infinite dimension. Since the error is identically independent distributed, we use LS-C information criterion as the selection criterion for the number of clusters, where q(M)=MK, $A_{n}=clog\left(n\right)$ where c=2. Adopting the FCR-HL model proposed in this paper, the information criterion is obtained as shown in Figure 1:

Figure 1 shows that LS-C reaches the minimum when K=3 (the scale of the vertical axis is so large that the values of LS-C at K=3 and K=2 in Figure seem to be close, but they are not), which is the estimation of the optimal number of clusters and consistent with the number of real clusters. When K=1, the LS-C reaches the maximum which means that data without partition have poor performance. It encourages us to pay more attention to the clustering regression. To show the superiority of the FCR-HL model, we compare the performance of the confusion matrix with that of the K-means that is a popular and widely used cluster method in Table 1 and Table 2.

Each row in a confusion matrix represents a predicted cluster, while each column represents a real cluster. Table 1 shows that 440, 477, and 490 samples were correctly clustered into groups, respectively. The confusion matrix of the K-means clustering method in Table 2 indicates that the K-means method has a good behavior on the partition of the first group, but it cannot effectively partition the data of the second and third group. Considering the relationship between response variables and functional explanatory variables, our model shows how the regression relationships change between clusters, not the distance of the data observations themselves. The confusion matrixes show the improvement when the auxiliary information has been added into the partition process of the data. Next, we show the heterogeneity between the clusters.

#### 3.1.2. Heterogeneous Hidden Information Mining

Traditional cluster methods, such as the K-means method, can only provide partition results, while our model can provide regression information of each cluster. In addition, when the regression analysis is carried out to the different clusters, the heterogeneity can be mined. If the regression analysis is carried out to the data without partition, the heterogeneity has been ignored and the results of the regression analysis may be exact enough or even wrong. Therefore, it is necessary to identify the heterogeneity of data with the help of FCR-HL model.

First, the regression results of the data without partition are shown in Table 3.

Table 3 shows that if the data has not been clustered, that is, assuming that all the data are from one population, the estimated parameters are all significant at the significance level 0.05, while the $R^{2}$ that explains the goodness of fitting is only 0.3371, which is relatively small. Again, it is necessary to identify the heterogeneity of the data first, and then perform regression analysis within clusters. Then, the results will be more reliable.

Using the FCR-HL model in the simulated data by setting d=0.02, which is adjusted adaptively, we have the regression results of the three clusters. And the regression results of three clusters are shown in the Table 4.

The parameters in the three groups are all significant at the significance level of 0.05, and the $R^{2}$ of the three groups are 0.9158, 0.9994 and 0.9981, respectively, which means that these three regressions have better performance. Therefore, it can be shown that the FCR-HL model has improved the fitting effect and obtain reliable parameter estimations.

Comparing with the results given by the K-means method, our model utilizes the relationship between the response variable and the functional explanatory variable, and incorporates this auxiliary information into the cluster process to improve the accuracy of clustering. In addition, the FCR-HL model can update the parameter estimates by updating the principal component scores and the number of clusters when new data enters into the sample.

### 3.2. Climate Data

**Figure 2:**
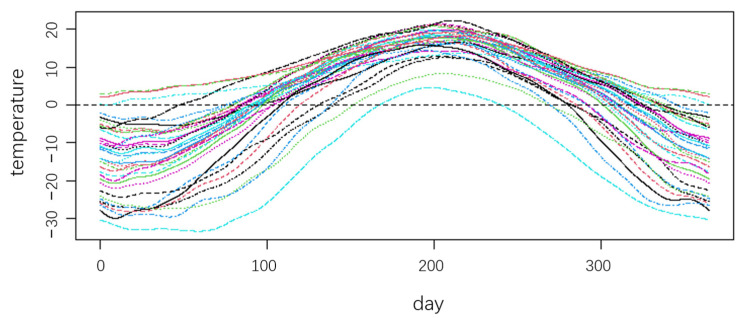
Temperature data for 35 sites (one color stands for one site).

**Figure 3:**
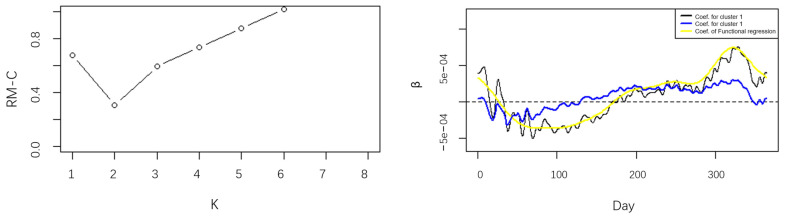
RM-C values over the candidate K (the **left** panel) and coefficients for both the pooled data and the clustered data (the **right** panel).

Using the classic Canadian weather data, which contains the annual temperature change and rainfall information of 35 stations, the annual rainfall is used as the response variable, using the temperature as the explanatory variable to study the influence of temperature on rainfall. Figure 2 shows the temperature of each site:

From Figure 2, although the temperature at each site has a similar trend of change, there are differences in the size of the trend change and the time of the change. If we disregard these characteristics and do regression analysis on the pooled data directly, the finding may be contradictory to the truth. Moreover, the relationship between the temperature and rainfall is very important to distinguish which cluster the data belongs to. We use the FCR-HL model to partition the data and explain the impact of temperature on rainfall. The number of sites is small, the maximum likelihood parameter estimation method is no longer applicable, and the robust estimation algorithm will be used to estimate the parameters. Thus, the RM-C is calculated to obtain the optimal number K and the cluster results shown in Figure 3.

The left picture of Figure 3 is the value of RM-C under different K obtained by the iterative algorithm, and it is obvious that RM-C reaches the minimum at K=2, which means that the optimal number of clusters is 2. The sites belonging to the two clusters are shown in Table A3, which shows that the logarithmic annual rainfall of the sites in cluster1 are always bigger than sites in cluster2. This cluster result is convincing.

To clearly illustrate the usefulness of the FCR-HL model, the parameter estimations for the two clusters given by the FCR-HL model and parameter estimations given by the direct regression on the pooled data. The parameter beta in the picture explains the impact of the temperature on annual rainfall over time. As we can see, although the yellow curve obtained by the use of regression analysis on the pooled data is smother than both the black curve and the blue curve, which corresponds to the cluster1 and cluster2, respectively, and are obtained by the FCR-HL model, the latter two curves can better reflect the considerable difference fluctuation of the data. That is, the parameter estimations after clustering can highlight the different characteristics of the data from different sub-populations. More specifically, the parameter estimations for the cluster1 are bigger than the parameter estimations for the cluster2 when t≤50 and t≥230, which indicates that the influence of temperature on the annual rainfall of the sites in cluster1 is always bigger than that of the sites in the cluster2, and the parameter estimations of the cluster1 are smaller than the parameter estimations of the cluster2 when 50≤t≤230, which indicates that the influence of temperature on the annual rainfall is always smaller for the sites of cluster1 than the sites of cluster2, conversely. In addition, the black curve and blue curve have shown considerable different degrees of volatility, which indicates that the influence of temperature on annual rainfall varies between groups and also over time.

In addition, Table 5 represents the parameter estimations of the regression directly on the pooled data. Cluster1 and cluster2 stand for the regression on the partitioned data. The *R*^2^ value of the pooled data and two clusters are 0.7567, 0.5692 and 0.6868, respectively. It is noted that there are only thirty-five sites, and the sample sizes of the two clusters are not large enough to ensure the unbiasedness and consistency of both the score estimators $\hat{\xi }$ and the regression estimators $\hat{\beta }$. Given this condition, the *R*^2^ value of the clustered data is not promoted. However, Table A3 shows the reasonability of the partitions given by FCR-HL model. Although the performance of the *R*^2^ value is not ideal for the Canadian weather data, we may still suggest adopting the proposed model in clustering and heterogeneity learning.

After analyzing the simulated data and climate data, it can be seen that the FCR-HL model improves the accuracy of clustering, and it can be seen from the results that the FCR-HL model can detect the heterogeneity information.

### 3.3. China Air Pollution Data

**Figure 4:**
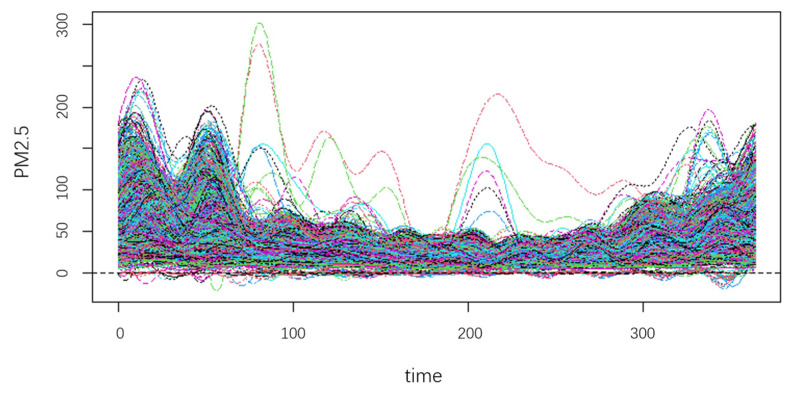
The PM_2.5_ concentrations at all sites (one color stands for one station).

As typical pollutants in atmosphere, inhalable particulate matter such as PM_10_ and PM_2.5_ bring risk to human health [1,30] and obtain a lot of attention from researchers. In the research on urban air quality, the concentration of PM_10_ and PM_2.5_ have a significant correlation with each other. This correlation has variability across seasons and regions. This paper uses the FCR-HL model to study the heterogeneity and characteristics of air quality in different regions of China. We first obtain national air quality data from China Meteorological Data Network (https://www.resdc.cn/data.aspx?DATAID=289, accessed on 15 February 2023), and then we clean the data. Finally, we obtain PM_10_ and PM_2.5_ concentration data of 1602 stations across the country from 1 January 2019, to 31 December 2019, with a frequency of hours. The annual average PM_10_ concentration of each station was obtained as a response variable, and the daily average PM_2.5_ concentration was obtained as a functional explanatory variable. Figure 4 shows the functional representation of discrete PM_2.5_ concentration data: 

Figure 4 shows that the PM_2.5_ concentration at some stations exhibit the same pattern of change over time, while there are different patterns at other stations in the same period. The coefficients of the regression are inexact if we make use of regression analysis on all the data, as different groups of stations possess different relationships between PM_10_ and PM_2.5_. This indicates that the stations need to be partitioned first. At the same time, the auxiliary information would be beneficial for clustering.

Using the FCR-HL model proposed in this paper and considering the auxiliary information between PM_10_ and PM_2.5_, we first perform functional principal component analysis on PM_2.5_, and build a functional regression model:

(28) $$y_{i}={\hat{\xi }}_{i}^{\prime }\beta +e_{i}$$

where $y_{i}$ is the PM_10_ concentration for the station i, and ${\hat{\xi }}_{i}$ is the vector estimated scores of the PM_2.5_ for the station i.

**Figure 5:**
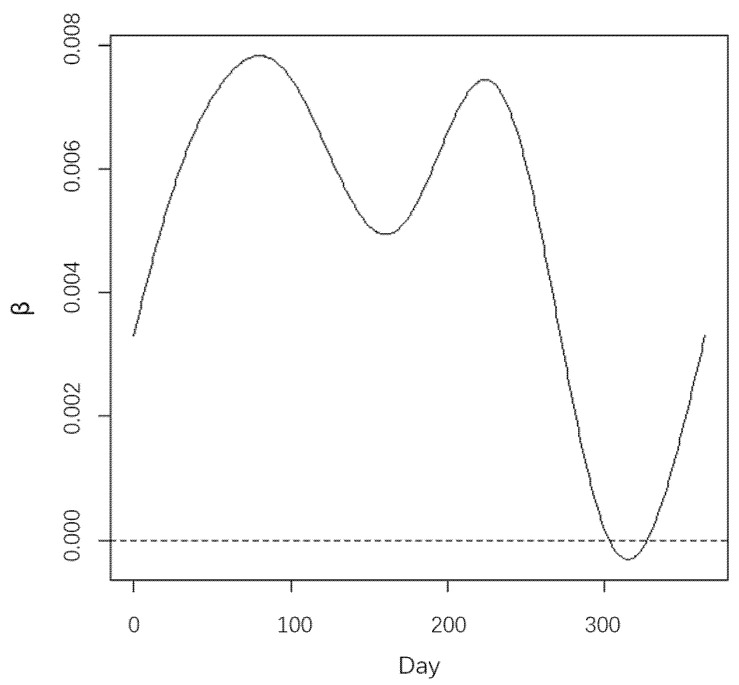
Regression parameter estimation without partitioning.

**Figure 6:**
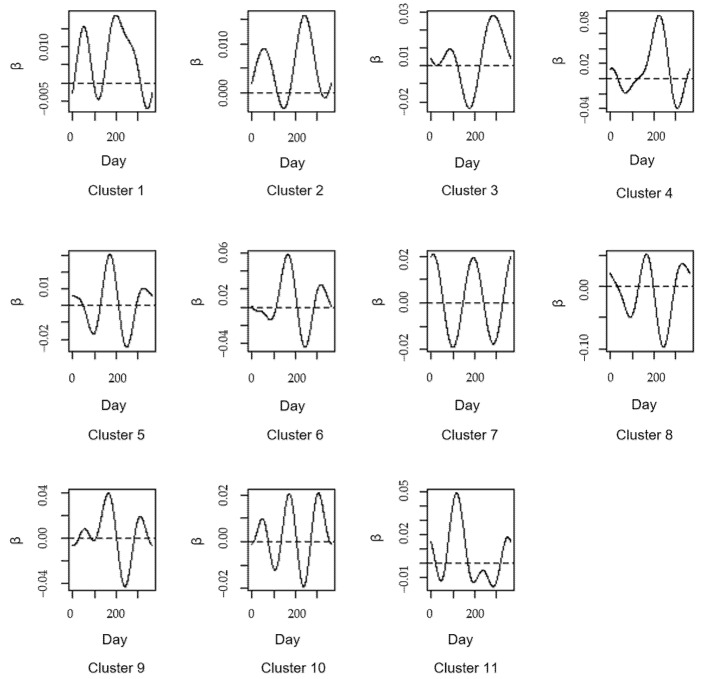
Parameter estimation of the different partitions given by the FCR-HL models.

To explain the advantages and necessity of the model more clearly, firstly, the coefficients over time of the functional regression without partitioning are shown in Figure 5, and then the coefficients over time of the functional regression in each cluster are shown in Figure 6.

The coefficients in Figure 5 and Figure 6 show how the impact of PM_2.5_ on PM_10_ changes over time without partitioning and under partitions, respectively. The coefficient indicates the annual average change in PM_10_ concentration with daily PM_2.5_ concentration, and their signs indicate the positive and negative aspects of the correlation. Note that the impact of PM_2.5_ on PM_10_ means that the PM_10_ concentration changes with the change of the PM_2.5_ concentration over time. In Figure 6, according to the iterative clustering algorithm, the stations are divided into 11 groups as the optimal number of clusters is estimated to be 11, and the heterogeneity partition for analyzing the relationship between PM_10_ and PM_2.5_ are also shown.

As we can see, the regression coefficients in Figure 5 are much smoother over time. Most of them are small and positive values, which consider that the influence of PM_2.5_ on PM_10_ is almost positive and small over time for all stations. However, the impact may not be exact enough without taking into account the heterogeneities between stations as mentioned above. On the contrary, the coefficients after utilizing FCR-HL model are more convincing for the influence of PM_2.5_ on PM_10_. In Figure 6, the regression coefficients for all 11 groups have entirely different characteristics. First, these regression coefficients are both positive and negative, which differ from those in Figure 5. Second, the regression coefficients of all 11 clusters have more steep variation trends than those in Figure 5. Regression coefficients differ from one cluster to another. For example, the coefficient of cluster1 shows a rise and then a fall, and includes a local maximum and a local minimum from day 0 to day 100, which means that the impact of PM_2.5_ on PM_10_ increases first and then decreases, while the coefficient of cluster 5 only decreases and only has one local minimum from day 0 to day 100, which means that the impact of PM_2.5_ on PM_10_ is always decreasing. For another example, a similar trend is observed for the coefficients of cluster1 and cluster 9 from day 0 to day 100, but the values are different, especially the local maximum and local minimum. All these differences between the 11 clusters can demonstrate the existence of heterogeneity and the importance of the heterogeneity partition. Additionally, the coefficients of each cluster can better show the varying impact over time. As for the stations of the cluster1, the impact of PM_2.5_ on PM_10_ is negative at the beginning and then positive until close to day 100, from where the impact is negative until close to day 230 and then positive until day 300, and the impact is negative on the last day. By that analogy, how the impact of PM_2.5_ on PM_10_ varies over time and how much impact of PM_2.5_ on PM_10_ at a fixed time can be obtained for the 11 clusters.

In addition, from (28), the parameters estimations for the pooled data and grouped data are shown in Table A1 and Table A2 (in Appendix A), respectively. It can be seen from Table A1 that the functional principal component scores 5 and 6 in the regression results are not significant, and the$\;R^{2}$ of the regression is 0.7484. In Table A2, the results of the 11 groups divided by the FCR-HL model show that the functional principal component scores are almost significant except the score3 in the 4th group and the score6 in the 7th group, and the $R^{2}$ of all groups are all above 0.9. It is noted that the functional explanatory variable in the FCR-HL model is expanded by the Karhunen-loeve theory, after which the functional principal component scores contain the essential information of the explanatory variable. Thus, the insignificance of the functional principal component scores in Table A1 resulting in the rejection of important parameters bears witness to the inaccuracy of the regression analysis on the pooled data which has the heterogeneity feature. By contrast, almost all the parameters estimated by the FCR-HL model are significant, which means that almost all the essential auxiliary information has been widely used for clustering. Additionally, the improvement of $R^{2}$ of all the groups also testifies the efficiency of the FCR-HL model. In the end, these comparisons show that the FCR-HL model can effectively work on heterogeneity partition and mining its internal information.

The K-means method is also used to compare the performance of the $R^{2}$ with that of the FCR-HL model. The comparison is shown in Table 6.

From Table 6, it is obvious that FCR-HL model promote the goodness of the regression analysis of the PM_10_ on PM_2.5_ significantly. Considering the auxiliary information will improve the cluster results, since the distance that is the key role in clustering has been set to be the distance between the data and regression hyperplane, not between the data itself. Then, the results of the FCR-HL model help us to better understand how the PM_2.5_ concentrations impact the PM_10_ concentrations.

In summary, the PM_2.5_ and PM_10_ data in the empirical analysis section are clustered into 11 groups by using our model. On one hand, the impact of the PM_2.5_ on the PM_10_ varies over time and between groups, by which there is obvious heterogeneity between groups. On the other hand, the significance of the parameters in Table A1 and Table A2 expresses the importance of the partition to avoid the loss of essential information. In each cluster, the impact of the PM_2.5_ on PM_10_ has up-and-down fluctuations, which means that the PM_10_ concentration changes with the PM_2.5_ concentration up and down. Moreover, the coefficients in all clusters show that the impact is not always positive.

## 4. Discussion

This paper constructs a heterogeneity learning model from the perspective of data clustering, which can solve the problem of clustering and provide implicit structural information about heterogeneity at the same time. Combining the regression model with the clustering algorithm can not only incorporate more effective information into the clustering and improve the accuracy of the clustering, but also analyze more precisely the relationship between the explanatory variables and the explained variables in different clusters (also called as the subpopulations). In addition, because the complexity of the actual data makes the classical regression model unable to capture the continuous characteristics of the data, we need functional data analysis techniques to add the continuous characteristics of the data to improve the research of regression clustering. Based on the functional data analysis technology, this paper uses the principal component scores and then reconstructs a new regression function. Using the iterative algorithm and information criterion, we can obtain the number of clusters and parameter estimations simultaneously. Regarding the FCR-HL model, the advantages in statistics are: first, each parameter estimation is consistent; second, the iterative algorithm can give cluster results at the same time, and they can be updated when new samples are added. The advantage in the application is that it detects the heterogeneity in data and explains how the covariate impacts the response.

In addition, both data simulation and empirical results illustrate the effectiveness of the new model and its broad application prospects. Simulation, case data and empirical data are used, and the results given by the FCR-HL model are compared with the that of the well-known K-means method. The comparisons show that our model can better partition data as the regression clustering utilizes the auxiliary information to explain how differently regression performs across the clusters, while the K-means method focus on how the distances among data behave differently across the clusters.

In summary, the data for environmental research and public health, for example, the climate data, has heterogeneity. In this way, the FCR-HL model is proving to be hugely powerful. Two future directions are pointed out. (1) We will analyze other types of air pollution data and public health data to make our model more systematized and comprehensive. (2) The auxiliary information plays an important role in our model, and data is inextricably linked in a complex social network. Therefore, digging more useful information, such as the network information and text information, and then putting them into the model will improve the study of the heterogeneity learning.

## 5. Conclusions

In this paper, we proposed the FCR-HL model to handle air pollution data. Firstly, the starting point of the article lies in the heterogeneity learning in the data and extends it to the functional data. Secondly, we introduce the model design, parameter estimation, and the iterative algorithm. To testify the validity of our model, the famous K-means and our model are both used in the simulation and climate data, and the performance of our model is better. An empirical analysis on the air pollution data is adopted in the final. The results show that the impact of the PM_2.5_ on PM_10_ varies between clusters and over time. To sum up, the FCR-HL model captures the continuity in data itself and incorporates the auxiliary information to support multiple pieces of information, including the number of subpopulations and how the PM_2.5_ impact the PM_10_ over time. Thus, this model may provide some effective information for the policymaking department and a new perspective for research.

## Figures and Tables

**Table 1:** Confusion Matrix of the simulation data based on the FCR-HL model.

			Real Cluster	
		Cluster1	Cluster2	CLUSTER3
Predicted cluster	cluster1	440	9	8
	cluster2	19	477	2
	cluster3	41	14	490

**Table 2:** Confusion Matrix of the simulation data based on the K-means model.

			Real Cluster	
		Cluster1	Cluster2	Cluster3
Predicted cluster	cluster1	500	246	241
	cluster2	0	129	127
	cluster3	0	125	132

**Table 3:** Regression Results for the Pooled Data.

	Estimate	Std. Error	*t* Value	Pr(>|t|)	
(Intercept)	−0.03764	0.28243	−0.133	0.894	
Score1	0.22366	0.02075	10.781	<2 × 10^−16^	***
Score2	−1.27693	0.05027	−25.401	<2 × 10^−16^	***

Significance: ‘***’ represent significant at the significance level of 0.

**Table 4:** Regression Results based on the FCR-HL Model.

		Estimate	Std. Error	*t* Value	Pr(>|t|)	
cluster1	(Intercept)	0.0538	0.0208	2.584	0.0101	*
	Score1	0.3107	0.0049	63.228	<2 × 10^−16^	***
	Score2	0.3525	0.0087	40.321	<2 × 10^−16^	***
cluster2	(Intercept)	−0.0704	0.0222	−3.166	0.00164	**
	Score1	3.2098	0.0037	858.3	<2 × 10^−16^	***
	Score2	−2.1598	0.0069	−312.43	<2 × 10^−16^	***
cluster3	(Intercept)	−0.0782	0.0201	−3.899	0.000109	***
	Score1	0.0169	0.0009	18.151	<2 × 10^−16^	***
	Score2	−1.2642	0.0024	−536.697	<2 × 10^−16^	***

Significance: ‘***’, ‘**’, ‘*’ represent significant at the significance level of 0, 0.001 and 0.01, respectively.

**Table 5:** The regression results for both the pooled data and the clustered data given by the FCR-HL model.

		Estimate	Std. Error	*t* Value	Pr(>|t|)	
pool data	(Intercept)	2.8148	0.0252	111.6310	0.0000	***
	score1	0.0016	0.0002	7.9700	0.0000	***
	score2	0.0016	0.0007	2.4350	0.0211	*
	score3	−0.0062	0.0014	−4.4100	0.0001	***
	score4	−0.0054	0.0027	−1.9860	0.0562	.
group1	(Intercept)	2.8476	0.0729	39.0860	2 × 10^−16^	***
	score1	0.0017	0.0009	1.9420	0.0711	
	score2	0.0021	0.0010	2.0300	0.0605	*
	score3	−0.0061	0.0022	−2.7980	0.0135	*
	score4	−0.0129	0.0085	−1.5110	0.1516	
group2	(Intercept)	2.5803	0.0421	61.2410	0.0000	***
	score1	0.0004	0.0003	1.4130	0.1879	
	score2	−0.0012	0.0007	−1.8560	0.0931	
	score3	−0.0029	0.0015	−1.8790	0.0896	
	score4	−0.0045	0.0020	−2.2300	0.0498	*

Significance: ‘***’, ‘*’, ‘.’, and ‘ ’ represent significant at the significance level of 0, 0.01, 0.05, and 1, respectively.

**Table 6:** The performance of the $R^{2}$ of the K-means method and FCR-HL model.

	Cluster	$R^{2}$
K-means	cluster1	0.1761
	cluster2	0.5314
	cluster3	0.1686
	cluster4	0.7081
	cluster5	0.4111
	cluster6	0.7845
FCR-HL model	cluster1	0.9939
	cluster2	0.9947
	cluster3	0.9939
	cluster4	0.9734
	cluster5	0.9723
	cluster6	0.9941
	cluster7	0.9873
	cluster8	0.9916
	cluster9	0.9893
	cluster10	0.9955
	cluster11	0.9983

## Appendix A

**Table A1:** The regression results of the pooled air pollution data.

	Estimate	Std. Error	*t* Value	Pr(>|t|)	
(Intercept)	63.6477	0.4041	157.519	2 × 10^−16^	***
score1	0.0781	0.0012	67.745	2 × 10^−16^	***
score2	−0.0446	0.0046	−9.764	2 × 10^−16^	***
score3	0.0479	0.0067	7.201	9.15 × 10^−13^	***
score4	1.2418	0.5128	2.422	0.0156	*
score5	−0.8467	0.5523	−1.533	0.1255	
score6	1.9677	2.25668	0.872	0.3834	

Significance: ‘***’, ‘*’, and ‘ ’ represent significant at the significance level of 0, 0.01, and 1, respectively.

**Table A2:** Regression results of the clustered air pollution data given by the FCR-HL model.

		Estimate	Std. Error	*t* Value	Pr(>|t|)	
cluster1	(Intercept)	6.45 × 10	1.76 × 10^−1^	365.78	<2 × 10^−16^	***
	score1	8.81 × 10^−2^	5.88 × 10^−4^	149.88	<2 × 10^−16^	***
	score2	−7.03 × 10^−2^	2.64 × 10^−3^	−26.59	<2 × 10^−16^	***
	score3	5.82 × 10^−2^	3.06 × 10^−3^	19	<2 × 10^−16^	***
	score4	6.59	2.34 × 10^−1^	28.22	<2 × 10^−16^	***
	score5	−4.3	2.17 × 10^−1^	−19.8	<2 × 10^−16^	***
	score6	−1.44 × 10	1.11	−12.96	<2 × 10^−16^	***
cluster2	(Intercept)	55.0751	0.1337	411.858	<2 × 10^−16^	***
	score1	0.0789	0.0005	160.485	<2 × 10^−16^	***
	score2	-0.05	0.0019	−26.169	<2 × 10^−16^	***
	score3	0.0058	0.0023	2.573	0.011	*
	score4	3.3271	0.1821	18.266	<2 × 10^−16^	***
	score5	−3.9563	0.2132	−18.555	<2 × 10^−16^	***
	score6	2.1775	0.7535	2.89	0.0044	**
cluster3	(Intercept)	60.4412	0.2281	264.932	<2 × 10^−16^	***
	score1	0.0974	0.0008	123.178	<2 × 10^−16^	***
	score2	−0.0468	0.0032	−14.712	<2 × 10^−16^	***
	score3	−0.161	0.0032	−50.531	<2 × 10^−16^	***
	score4	−6.1258	0.2505	−24.454	<2 × 10^−16^	***
	score5	−7.8931	0.2726	−28.958	<2 × 10^−16^	***
	score6	6.3206	1.1952	5.288	5.50 × 10^−7^	***
cluster4	(Intercept)	104.0814	2.0545	50.661	<2 × 10^−16^	***
	score1	0.0315	0.0053	5.981	1.30 × 10^−6^	***
	score2	−0.259	0.0148	−17.474	<2 × 10^−16^	***
	score3	0.0167	0.0291	0.574	0.57	
	score4	20.1699	1.6918	11.922	4.11 × 10^−13^	***
	score5	7.6024	1.6622	4.574	7.26 × 10^−5^	***
	score6	52.44	6.3217	8.295	2.27 × 10^−9^	***
cluster5	(Intercept)	6.12 × 10	1.62 × 10^−1^	376.952	<2 × 10^−16^	***
	score1	3.28 × 10^−2^	7.63 × 10^−4^	42.908	<2 × 10^−16^	***
	score2	3.97 × 10^−2^	2.02 × 10^−3^	19.622	<2 × 10^−16^	***
	score3	−2.03 × 10^−2^	2.40 × 10^−3^	−8.432	2.08 × 10^−14^	***
	score4	9.82 × 10^−1^	2.37 × 10^−1^	4.151	5.41 × 10^−5^	***
	score5	9.92	2.71 × 10^−1^	36.566	<2 × 10^−16^	***
	score6	−2.99 × 10	9.93 × 10^−1^	−30.08	<2 × 10^−16^	***
cluster6	(Intercept)	6.55 × 10	1.58 × 10^−1^	415.781	<2 × 10^−16^	***
	score1	6.05 × 10^−2^	6.30 × 10^−4^	96.018	<2 × 10^−16^	***
	score2	−9.49 × 10^−2^	2.13 × 10^−3^	−44.605	<2 × 10^−16^	***
	score3	1.09 × 10^−2^	2.10 × 10^−3^	5.194	6.6 × 10^−7^	***
	score4	−7.36	1.80 × 10^−1^	−40.973	<2 × 10^−16^	***
	score5	1.49 × 10^−1^	2.27 × 10^−1^	65.489	<2 × 10^−16^	***
	score6	−5.20 × 10	9.65 × 10^−1^	−53.847	<2 × 10^−16^	***
cluster7	(Intercept)	74.7728	0.2767	270.233	<2 × 10^−16^	***
	score1	0.0388	0.001	39.286	<2 × 10^−16^	***
	score2	0.1413	0.0031	45.309	<2 × 10^−16^	***
	score3	−0.0197	0.0042	-4.719	7.42 × 10^−6^	***
	score4	10.5498	0.2731	38.627	<2 × 10^−16^	***
	score5	9.7765	0.3227	30.291	<2 × 10^−16^	***
	score6	0.5924	1.7418	0.34	0.734	
cluster8	(Intercept)	77.3713	0.3616	213.98	<2 × 10^−16^	***
	score1	−0.0449	0.0015	−30.61	<2 × 10^−16^	***
	score2	0.3551	0.0048	74.57	<2 × 10^−16^	***
	score3	−0.1436	0.0059	−24.25	<2 × 10^−16^	***
	score4	−7.0449	0.48	−14.68	<2 × 10^−16^	***
	score5	29.8419	0.5953	50.13	<2 × 10^−16^	***
	score6	−70.2609	2.4218	−29.01	<2 × 10^−16^	***
cluster9	(Intercept)	6.29 × 10	1.69 × 10^−1^	372.187	<2 × 10^−16^	***
	score1	5.10 × 10^−2^	6.71 × 10^−4^	75.997	<2 × 10^−16^	***
	score2	−2.14 × 10^−2^	2.41 × 10^−3^	−8.901	2.45× 10^−15^	***
	score3	9.14 × 10^−2^	2.57 × 10^−3^	35.527	<2 × 10^−16^	***
	score4	−6.33	2.25 × 10^−1^	−28.095	<2 × 10^−16^	***
	score5	5.51	2.26 × 10^−1^	24.375	<2 × 10^−16^	***
	score6	−5.27 × 10	1.03 × 10	−51.265	<2 × 10^−16^	***
cluster10	(Intercept)	6.10 × 10	1.15 × 10^−1^	530.873	<2 × 10^−16^	***
	score1	7.02 × 10^−2^	3.82 × 10^−4^	183.837	<2 × 10^−16^	***
	score2	1.00 × 10^−2^	1.53 × 10^−3^	6.573	4.07× 10^−10^	***
	score3	−3.93 × 10^−2^	2.21 × 10^−3^	−17.808	<2 × 10^−16^	***
	score4	−5.85 × 10^−1^	1.59 × 10^−1^	−3.684	0.0003	***
	score5	9.67 × 10^−1^	1.61 × 10^−1^	6.013	8.35 × 10^−9^	***
	score6	−4.00× 10	7.40 × 10^−1^	−54.035	<2 × 10^−16^	***
cluster11	(Intercept)	7.46 × 10	1.81 × 10^−1^	411.65	<2 × 10^−16^	***
	score1	9.24 × 10^−2^	4.03 × 10^−4^	229.33	<2 × 10^−16^	***
	score2	−9.89 × 10^−2^	2.14 × 10^−3^	−46.2	<2 × 10^−16^	***
	score3	1.28 × 10^−1^	3.33 × 10^−3^	38.49	<2 × 10^−16^	***
	score4	−1.28 × 10	2.44 × 10^−1^	−52.26	<2 × 10^−16^	***
	score5	8.20	2.68 × 10^−1^	30.61	<2 × 10^−16^	***
	score6	4.40 × 10	9.94 × 10^−1^	44.22	<2 × 10^−16^	***

Significance: ‘***’, ‘**’, ‘*’, and ‘ ’ represent significant at the significance level of 0, 0.01, and 1, respectively.

**Table A3:** Two clusters for the Canadian weather case by using the FCR-HL model.

	Sites
cluster1	St. Johns(3.17), Halifax(3.16), Sydney(3.17), Yarmouth(3.10), Charlottvl(3.08), Fredericton(3.05), Scheffervll(2.90), Arvida(2.95), Bagottville(2.97), Quebec(3.08), Sherbrooke(3.05), Montreal(2.97), Ottawa(2.96), Toronto(2.89), London(2.98), Thunderbay(2.85), Vancouver(3.06), Victoria(2.93), Pr. George(2.78), Pr. Rupert(3.41)
cluster2	Winnipeg(2.71), The Pas(2.65), Churchill(2.61), Regina(2.57), Pr. Albert(2.61), Uranium Cty(2.56), Edmonton(2.67), Calgary(2.60), Kamloops(2.43), Whitehorse(2.43), Dawson(2.52), Yellowknife(2.43), Iqaluit(2.62), Inuvik(2.42), Resolute(2.16)

The values in the brackets are the logarithmic annual rainfall of the sites.
